# Application of deep learning to predict the low serum albumin in new hemodialysis patients

**DOI:** 10.1186/s12986-023-00746-z

**Published:** 2023-04-24

**Authors:** Cheng-Hong Yang, Yin-Syuan Chen, Jin-Bor Chen, Hsiu-Chen Huang, Li-Yeh Chuang

**Affiliations:** 1 Department of Information Management, Tainan University of Technology, Tainan, Taiwan; 2 Department of Electronic Engineering, National Kaohsiung University of Science and Technology, Kaohsiung, Taiwan; 3 Program in Biomedical Engineering, Kaohsiung Medical University, Kaohsiung, Taiwan; 4 School of Dentistry, Kaohsiung Medical University, Kaohsiung, Taiwan; 5 Drug Development and Value Creation Research Center, Kaohsiung Medical University, Kaohsiung, Taiwan; 6 Department of Internal Medicine, Kaohsiung Chang Gung Memorial Hospital and Chang Gung University College of Medicine, Kaohsiung, Taiwan; 7 Department of Community Health, Chia-Yi Christian Hospital, Chia-Yi City, Taiwan; 8 Department of Chemical Engineering and Institute of Biotechnology and Chemical Engineering, I-Shou University, Kaohsiung, Taiwan

**Keywords:** Hemodialysis, Serum albumin, Grasshopper optimization algorithm, Quantile g-computation, Deep learning

## Abstract

**Background:**

Serum albumin level is a crucial nutritional indicator for patients on dialysis. Approximately one-third of patients on hemodialysis (HD) have protein malnutrition. Therefore, the serum albumin level of patients on HD is strongly correlated with mortality.

**Methods:**

In study, the data sets were obtained from the longitudinal electronic health records of the largest HD center in Taiwan from July 2011 to December 2015, included 1,567 new patients on HD who met the inclusion criteria. Multivariate logistic regression was performed to evaluate the association of clinical factors with low serum albumin, and the grasshopper optimization algorithm (GOA) was used for feature selection. The quantile g-computation method was used to calculate the weight ratio of each factor. Machine learning and deep learning (DL) methods were used to predict the low serum albumin. The area under the curve (AUC) and accuracy were calculated to determine the model performance.

**Results:**

Age, gender, hypertension, hemoglobin, iron, ferritin, sodium, potassium, calcium, creatinine, alkaline phosphatase, and triglyceride levels were significantly associated with low serum albumin. The AUC and accuracy of the GOA quantile g-computation weight model combined with the Bi-LSTM method were 98% and 95%, respectively.

**Conclusion:**

The GOA method was able to rapidly identify the optimal combination of factors associated with serum albumin in patients on HD, and the quantile g-computation with DL methods could determine the most effective GOA quantile g-computation weight prediction model. The serum albumin status of patients on HD can be predicted by the proposed model and accordingly provide patients with better a prognostic care and treatment.

## Introduction

The prevalence of end-stage renal disease (ESRD) has been continually increasing in various countries. According to a 2020 US Renal Data System report, Taiwan ranks among the top five countries globally in terms of the incidence rate of ESRD per million population. ESRD is a condition in which a person’s renal function declines to < 15% of normal renal function [1]. Patients with ESRD experience the symptoms of uremia, including loss of appetite, nausea, vomiting, itchy skin, facial and limb edema, and foul breath [2, 3]. Therefore, dialysis is required to alleviate symptoms and improve the quality of life of patients with ESRD [4]. Hemodialysis (HD) can effectively eliminate toxins and excess water from the kidneys. Patients with ESRD are required to undergo HD in a hospital two to three times per week throughout their life. In addition, receiving HD adversely affects patients’ quality of life and requires them to maintain diet control in terms of potassium, phosphorus, salt, water, and protein intake [5, 6]. Although HD can prolong patients’ lives, it may cause other complications, such as hypotension, hypertension, nausea, and vomiting, which may affect their physiological function and quality of life [7, 8]. Therefore, appropriate care and diet control are crucial for patients on HD [9]. Malnutrition may lead to increased mortality in patients on HD, and serum albumin level is a vital nutritional indicator for these patients [5, 10, 11]. The nutritional status of patients on HD is closely related to their clinical parameters, most represented by serum albumin, which may affect their risk of mortality [12]. To effectively prolong the survival of patients on HD, their clinical parameters should be maintained at normal levels.

Many related risk factors affect patients’ disease status, and appropriate medical care based on all possible risk factors cannot be currently provided. Therefore, the identification of the most crucial risk factors for diseases based on numerous biomarkers is essential. Most previous studies on this topic have recommended consultations with relevant disease specialists and the identification of risk factors for diseases; research and analysis should then be conducted by specialists [13]. Currently, machine learning (ML) methods have been widely used for disease diagnosis and prognosis, including artificial neural networks (ANN) [14], particle swarm optimization (PSO) [15], biogeography-based optimization [16, 17], and other hybrid technologies [18]. Previously, traditional statistical methods were used to compare data. ML and deep learning (DL) have the advantages of high accuracy, reproducibility, and objectivity. One of the major limitations of conventional ML techniques is the requirement of sometimes complex processing (feature engineering) to extract the requisite discriminative features [19]. Therefore, significant domain knowledge and data processing expertise were required to train non-deep learning models. Deep learning, however, is adept at learning abstract features directly from the raw data. Different layers of the network automatically learn abstract features representative of the data. A single well-designed and well-trained network can yield state-of-the-art results across many applications, without the need for significant domain knowledge [20]. It is clear that deep learning is an extremely powerful tool for learning complex, cognitive problems. However, it is not a comprehensive tool for all healthcare analytics applications. Several past commentaries on deep learning for clinical applications touch on how data issues such as low volume, high sparsity, and poor quality can limit the efficacy of deep learning methods. We find that conventional ML tools can achieve comparable, if not better performance in this context despite the complex nature of the data. Although deep learning can be applied to many of these fairly standard problems, conventional ML methods may provide simpler, cheaper, and more useful method for data modeling. Thus, their use for medical diagnosis and prognosis can be beneficial [18]. Traditional regression analysis may be inadequate for dealing with large and complex clinical data [21]. Studies have combined traditional statistics with ML and optimization algorithms to propose effective nursing strategies for patients on HD.

A metaheuristic optimization algorithm is commonly used to solve global optimization problems [22]. This algorithm is mainly used for searches by simulating nature and human intelligence to achieve optimal solutions. Heuristic optimization algorithms were first proposed in 1960 and are mainly divided into four categories: evolution, swarm intelligence, human intelligence, and physics and chemistry. Nature-inspired metaheuristic algorithms based on crowd intelligence are the most commonly employed [23], including PSO, grey wolf optimization, and whale optimization algorithms. Many nature-inspired metaheuristic algorithms have been developed and used in combination with other methods to solve complex problems in various fields and obtain the most favorable solution.

The grasshopper optimization algorithm (GOA) is a novel metaheuristic algorithm used for global optimization [24]. The GOA simulates the behavior of locust swarms and applies it to challenging problems in structural optimization. Exploration and exploitation are the two main stages of nature-inspired algorithms. The goal of the GOA is to improve the convergence speed of a search target and avoid local optima. A deep neural network is a DL method in machine learning [25]. Through imitation of the biological nervous system, models with different architectures are established for multiple operations and training to develop the optimal and most effective prediction model [26, 27].

Studies have reported that the serum albumin level in patients on HD is highly correlated with mortality and is a crucial factor for predicting mortality [28, 29]. This study used the GOA to determine the most favorable combination of risk factors for predicting the low serum albumin levels. Because interference factors may affect data, we used the quantile g-computation method for weight adjustment. Finally, we used the DL method to identify the most effective prediction model. This model was used to predict the serum albumin status of new HD patients. The findings of this study can help develop comprehensive prognostic care and treatment strategies for improving the quality of life and survival of new HD patients.

## Methods

### Data sets

**Fig. 1:**
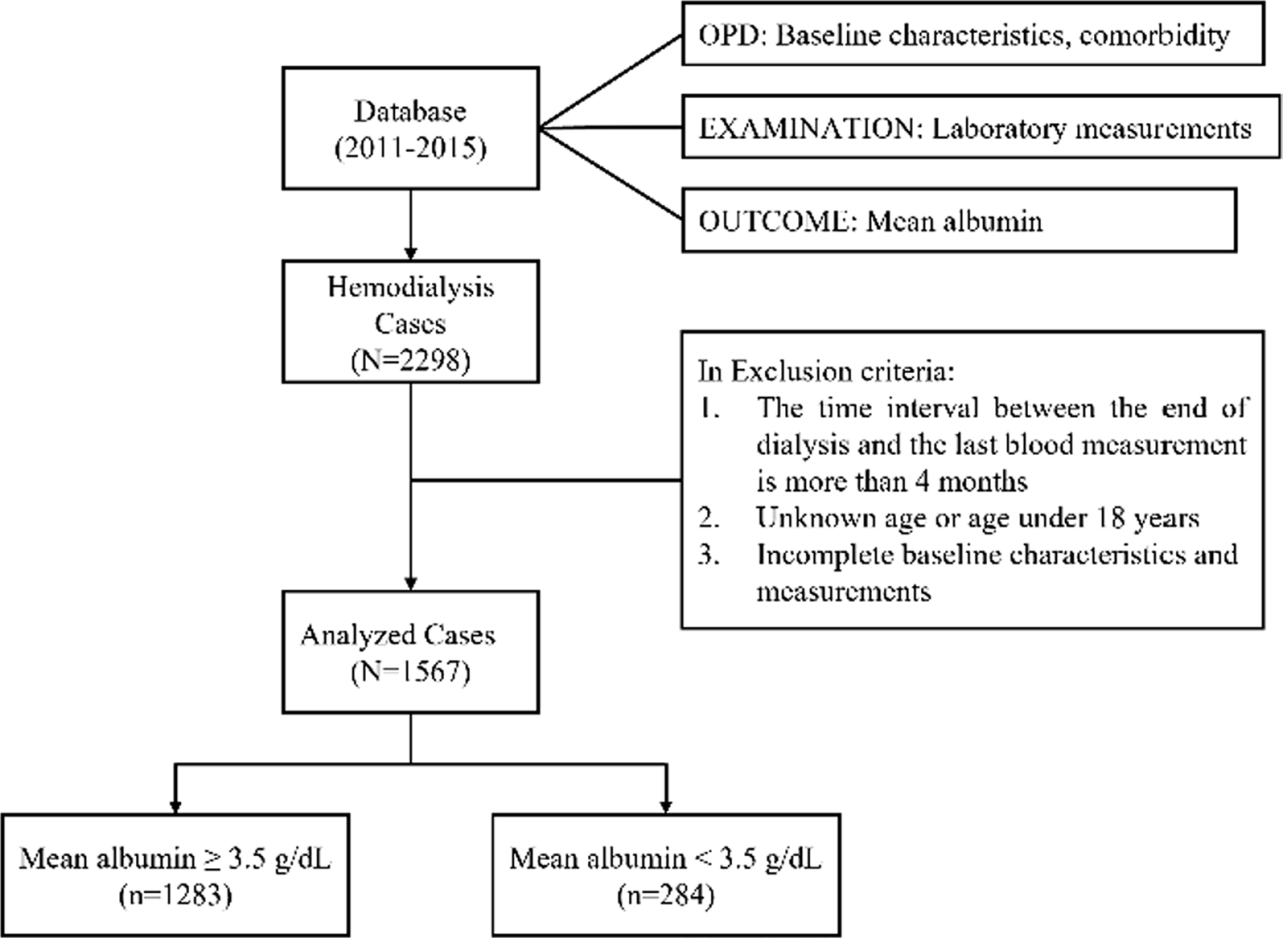
Data preprocessing workflow

This study used the data sets that were obtained from the longitudinal electronic health records of the largest HD center in Taiwan. A total of 2298 patients who received HD for more than 3 months and continued receiving HD three times a week from July 2011 to December 2015 were selected. We excluded the patients whose age was unknown, those aged < 18 years, those with a time interval of > 4 months between the end of dialysis and the last blood measurement, and those with incomplete data on baseline characteristics and laboratory measurements. Finally, we included 1567 patients who met the inclusion criteria in the analysis. All data were retrospectively collected using an approved data protocol (201800595B0), and the requirement for patients’ informed consent was waived. This study was conducted in accordance with the Declaration of Helsinki. Figure 1 presents the flowchart for the data processing.

Serum albumin level is strongly associated with mortality. This study identified the risk factors for a low serum albumin level and determined whether patients had a low serum level before death to predict mortality. To collect data on serum albumin levels, we recorded the levels monthly and calculated the mean by adding the levels measured three months before the study's end and three months before the patient's death. The standard used to classify serum albumin was 3.5 g/dL, which is based on Chang Gung Memorial Hospital's lower limit of the normal range in Taiwan. The patients were categorized into two groups: those with a mean albumin level ≥ 3.5 g/dL and those with a mean albumin level < 3.5 g/dL. In addition, we collected data on demographics; comorbidities; causes of mortality; and mean albumin level–related clinical laboratory data, namely age, gender, diabetes, hypertension, heart failure, cancer, and mortality status. Baseline laboratory parameters included hemoglobin, serum albumin, iron, ferritin, sodium, phosphate, blood urea nitrogen, creatinine, alkaline phosphatase, intact parathyroid hormone, cholesterol, triglyceride, and fasting glucose levels.

**Fig. 2:**
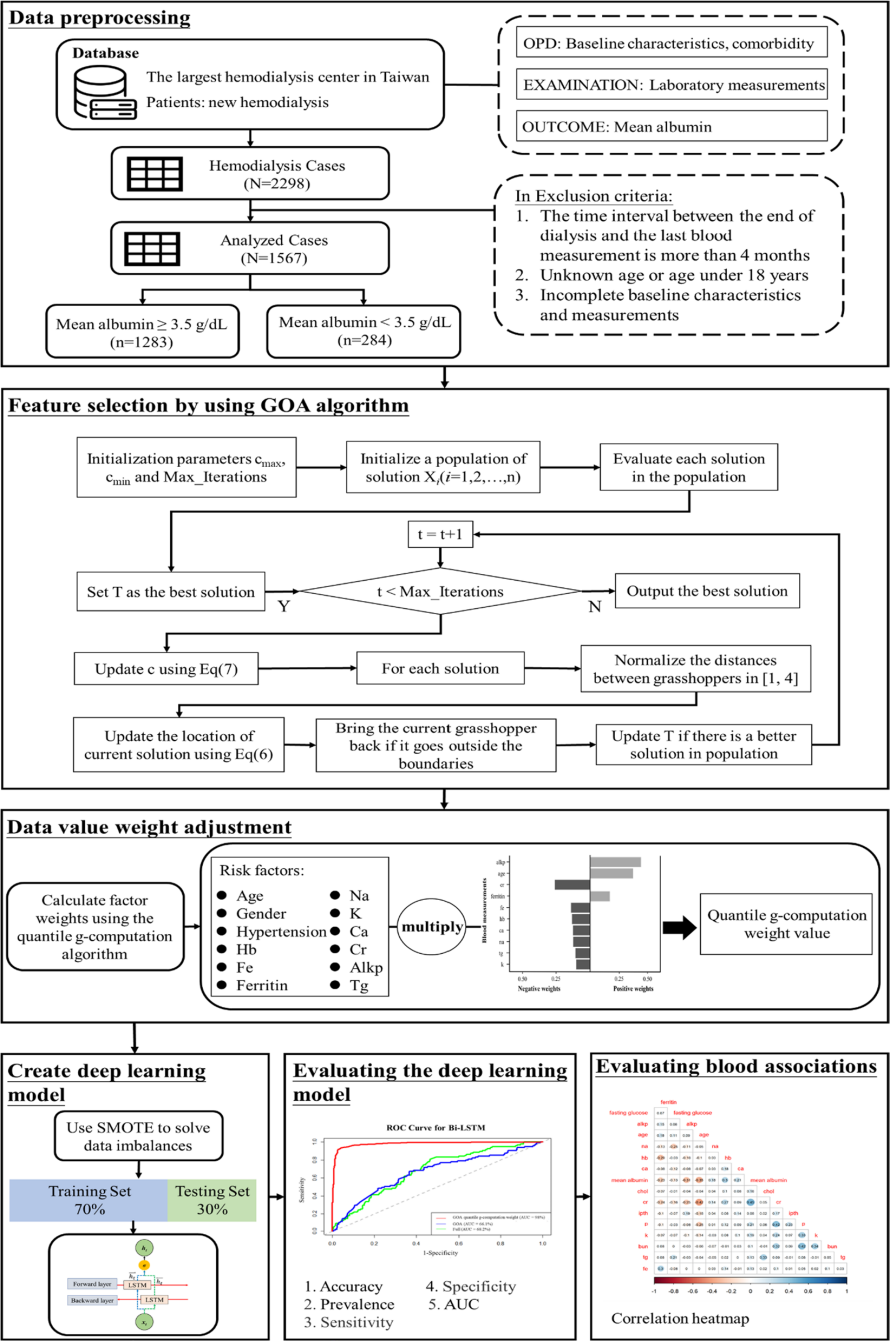
Analysis flowchart

Figure 2 illustrates the analytical workflow for predicting low serum albumin levels in patients on HD. In the first step, data were extracted from the longitudinal electronic health records of the largest HD center in Taiwan. We collected data on diagnosis, complications, and laboratory measurements. Subsequently, we cleaned, filtered, and merged the data. In the second step, we used the GOA for feature selection to determine the most favorable combination of risk factors for predicting low serum albumin levels. In the third step, we adjusted the weight of the data. The quantile g-computation method was used to examine the most favorable factor combinations selected using the GOA; this method enabled the ranking of the importance of risk factors for low serum albumin levels and the calculation of the positive and negative weights of each risk factor for low serum albumin levels condition. The weights were used to adjust blood levels such that they significantly differed from each other. In the fourth step, we established the prediction data. We used the synthetic minority oversampling technique to solve data imbalances. This method is based on the concept of the K-nearest neighbor (KNN). The data set was split into training and testing sets at a ratio of 7:3; these data were then used to establish a prediction model. Seven methods, namely the KNN, SVM, RF, GBDT, XGBoost, DNN, and Bi-LSTM, were used to establish three prediction models. In the fifth step, we evaluated the prediction model; plotted the receiver operating characteristic (ROC) curve of each model; and calculated the accuracy, prevalence, sensitivity, specificity, and area under the curve (AUC) to determine and compare the quality of the prediction models. In the sixth step, we evaluated the correlation between the clinical factors, drew a Pearson correlation diagram, and used a visual heatmap method to evaluate positive and negative correlations between blood parameters by visual.

### Grasshopper optimization algorithm (GOA)

The GOA, which was proposed by Saremi et al. in 2017, simulates the foraging behavior of grasshoppers [30]. Because of its high compatibility and ability to evaluate complex traits, the GOA has been used for the selection of multiple factors [31, 32]. The GOA can accelerate the integration of complex trait interactions among multiple factors. Moreover, the GOA can be used to solve various optimization problems, including engineering, computer, and feature selection problems [30]. The GOA is significantly superior to other classical algorithms, such as the PSO algorithm, the differential evolution (DE) algorithm, and the genetic algorithm (GA). In addition, the GOA can be used to manage different data sets [32]. The GOA can yield more favorable results and shorten calculation time of the criteria of fitness and average classification accuracy. In addition, the GOA can be combined with other methods to develop other hybrid GOA [33]. The accuracy and performance of the original algorithm can be improved, and these hybrid algorithms can be used in various fields. Therefore, we used a combination of the GOA and the bidirectional long short-term memory (Bi-LSTM) method to improve model performance. In this study, we established an optimal multifactor correlation model by using GOA-based feature selection methods to determine the relationship between albumin level and clinical factors in patients on HD and to identify the related risk factors for low serum albumin levels for prediction of mortality risks in patients on HD.

The grasshopper is a herbivorous insect that usually appears alone in nature. However, millions of grasshoppers gathered in a cluster can act as pests. They can damage crops and are thus a concern in the agricultural industry. The lifecycle of a grasshopper consists of three stages: egg, nymph, and adult. Grasshoppers can be found in swarms during the life stages of nymph or adult. Slow movement and small steps are the main characteristics of grasshopper swarms in the larval phase. By contrast, sudden and long-distance movements are characteristic of adult groups. Food source seeking is a crucial feature of grasshopper swarms. The GOA is inspired by nature. Exploration and exploitation are the two main stages of nature-inspired algorithms. The algorithm aims to increase the convergence speed of searching for targets and avoid local optima. Search agents tend to move locally in the search space during the exploitation process but are encouraged to move suddenly during the exploration process. Grasshoppers perform these two processes and naturally find their target (food source). The flight path of a group of grasshoppers is affected by three factors: social interaction ($S_{i}$), gravity force ($G_{i}$), and wind advection force ($A_{i}$).

The GOA-based feature selection was used to accelerate convergence and identify associated risk factors for low serum albumin levels in patients on HD. In Eq. (1) presents a simulation of the swarming behavior of grasshoppers.

1 $$X_{i} = r_{1} S_{i + } r_{2} G_{i + } r_{3} A_{i}$$

where $X_{i}$ defines the position of the *i*-th grasshopper, $S_{i}$ is the social interaction in Eq. (2), $G_{i}$ is the gravity force on the *i*-th grasshopper in Eq. (4), and $A_{i}$ is the wind advection in Eq. (5). To ensure random behavior, $r_{1}$, $r_{2}$, and $r_{3}$ are considered random numbers in the range [0, 1].

2 $$S_{i} = \mathop \sum \limits_{j = 1, j \ne i}^{N} s\left( {d_{ij} } \right)\widehat{{d_{ij} }}$$

where $d_{ij}$ is the distance between the* i*-th and *j-*th grasshopper, calculated as $d_{ij} = \left| {X_{i} - X_{j} } \right|$, and $\widehat{{d_{ij} }} = \frac{{X_{i} - X_{j} }}{{d_{ij} }}$ is a unit vector from the *i*-th grasshopper to the *j-*th grasshopper. $s$ is a function used to define the strength of the social force in Eq. (3) calculated as follows.

3 $$s\left( r \right) = fe^{{\frac{ - r}{l}}} - e^{r}$$

where *f* indicates the intensity of attraction, and *l* is the attraction length scale.

4 $$G_{i} = - g \times \widehat{{e_{g} }}$$

where *g* is the gravitational constant and $\widehat{{e_{g} }}$ is a unity vector toward the center of the earth.

5 $$A_{i} = u \times \widehat{{e_{w} }}$$

where *u* is a constant drift and $\widehat{{e_{w} }}$ is a unity vector in the direction of wind.

Nymph grasshoppers have no wings; thus, their movements are highly correlated with wind direction.

Equation (6) is used to determine the current position of the *i*-th grasshopper, the position of all other grasshoppers, and the position of the target (food source).

6 $$X_{i}^{d} = c\left( {\mathop \sum \limits_{{j = 1,{ }j \ne i}}^{N} c\frac{{ub_{d} - lb_{d} }}{2}s\left( {\left| {x_{j}^{d} - x_{i}^{d} } \right|} \right)\frac{{x_{j} - x_{i} }}{{d_{ij} }}} \right) + \widehat{{T_{d} }}$$

where $ub_{d}$ is the upper bound in the *D*^th^ dimension, $lb_{d}$ is the lower bound in the *D*^th^ dimension $s\left( r \right) = fe^{{\frac{ - r}{r}}} - e^{r} ,$ $\widehat{{T_{d} }}{ }$ is the value of the *D*^th^ dimension in the target (the most favorable solution obtained thus far), and *c* is a decreasing coefficient used to shrink comfort, repulsion, and attraction zones. *S* is similar to *S* in Eq. (1). However, the gravity component *G* is not considered, and the wind direction *A* is assumed to be toward the target $\widehat{{T_{d} }}$.

In Eq. (6), the adaptive parameter *c* is used twice to simulate the deceleration of the locust that approaches the food source and that eventually consumes it. With an increase in the number of iterations, the outer *c* is used to reduce the search range of the target grasshopper, whereas the inner *c* is used to reduce the effect of the attraction and repulsion between grasshoppers in proportion to the number of iterations. To balance exploration and exploitation, the parameter *c* needs to be reduced in proportion to the number of iterations.

7 $${\text{c}} = {\text{c}}_{max} - {\text{l}}\frac{{c_{max} - c_{min} }}{L}$$

where ${\text{c}}_{max}$ is the maximum value of parameter c, $c_{min}$ is the minimum value of parameter *c*, *l* is the current iteration number, and *L* is the maximum number of iterations.

### Quantile g-computation

Quantile g-computation is a new method used to estimate the combined effects of mixtures [34]. It was proposed by Keil et al. in 2020 [35]. Quantile g-computation is based on parametric, generalized linear models. This method combines the simplicity of weighted quantile sum (WQS) regression with the flexibility of g-computation to estimate causal effects. Its advantages are that it is computationally efficient and can estimate positive and negative weights. Quantile g-computation does not require the assumption of direction homogeneity. This method redefines the positive and negative weights when directional homogeneity does not hold. The basic model of quantile g-computation is a joint marginal structural model given by the following formula.

8 $$E\left( {Y^{{X_{q} }} {|}Z,{\uppsi },\eta } \right) = g\left( {{\uppsi }_{0} + {\uppsi }_{1} S_{q} + \eta Z} \right)$$

where *Y* denotes outcomes, *X* refers exposures, and *Z* denotes some other possible covariates (e.g., potential confounders).* g* (⋅) is the link function in a generalized linear model (e.g., the inverse logit function of the probability of *Y* = 1 in a logistic model), ${\uppsi }_{0}$ is the model intercept, $\eta$ is the model coefficient for a set of covariates, and $S_{q}$ is an index representing the joint value of exposure.

Quantile g-computation (by default) converts all exposures *X* to *X*_*q*_. *X*_*q*_ converts exposure *X* to discrete fractions such as 0, 1, and 2, etc. By default, each exposure has four quantile cutoff points with a uniform distribution. Thus, *X*_*q*_ = 0 means that *X* is below the 25th percentile observed for that exposure. The index $S_{q}$ means that all exposures are set to the same value (by default, discrete values are 0, 1, 2, and 3). Thus, the parameter ${\uppsi }_{1}$ quantifies the expected change in results given that all exposures that are simultaneously increased by a quantile are possibly adjusted for Z.

The quantile g-computation allows the estimation of both ${\uppsi }_{1}$ and weights when the directional homogeneity assumption holds, and when the directional homogeneity does not hold, it allows valid inferences to be made regarding the effects of the entire exposure mixture as well as individual contributions to that mixture. First, the quantile g-computation transforms the exposure *X*_*j*_ to discretize $X_{j}^{q}$ through quartiles. Next, a linear model is fitted (other confounders *Z* are omitted for notational simplicity, but they can also be included):

9 $$Y_{i} = \beta_{0} + \mathop \sum \limits_{j = 1}^{d} \beta_{j} + \begin{array}{*{20}c} q \\ {ji} \\ \end{array} + \varepsilon_{i}$$

Third, under the assumption of directional homogeneity, ψ is given as $\mathop \sum \limits_{j = 1}^{d} \beta_{j}$ ($\beta_{j}$ is the impact size of exposure *j*), and each exposure weight is given by *k*. Weights are defined as the sum to 1.0.

10 $$W_{k} = \beta_{k} l\mathop \sum \limits_{j}^{d} \beta_{j}$$

When directional homogeneity does not hold, quantile g-computation redefines weights as negative or positive, which are interpreted as the proportion of negative or positive partial effects due to a particular exposure, and positive and negative weights are defined as the sum of both to 1.0.

### Synthetic minority over-sampling technique (SMOTE)

SMOTE is a synthetically sampled synthetic data algorithm proposed by Chawla et al. in 2002 [36]. SMOTE is used to solve the problem of data class imbalance by combining the oversampling minority and undersampling majority classes to synthesize data. Class imbalance is a common problem in classifier model training and is often encountered in the medical field. Therefore, this method can be used to increase the number of predicted event samples to make the data easier to train. The following steps are involved in SMOTE: (1) Find the KNN to the positive individual $X_{i}$. (2) Randomly select one of the k neighbors called $X_{j}$; this neighbor is used to generate new samples. (3) Calculate the difference between $X_{i}$ and $X_{j}$ in $= X_{j} - X_{i}$. (4) Generate a random number $\eta$ between [0, -1]. (5) Generate a new sample point $X_{i}^{{\left( {new} \right)}} = X_{i} - \eta$. The data set was split into training and testing sets at a ratio of 7:3. Thus, training set has 1097 and testing set had 470 patients. In this study, SMOTE had been implemented in the training set. And training set was increasing to 1715 patients.

### K-nearest neighbor (KNN)

The KNN algorithm was proposed by Peterson in 2009 [37]. The KNN algorithm is among the most fundamental and simple classification methods and should be one of the first choices for a classification study when little or no prior knowledge is available on the distribution of data. KNN classification was developed to perform discriminant analysis when the reliable parametric estimates of probability densities are unknown or difficult to determine. The traditional KNN method search an entire set of training data samples to classify an input test sample. Thus, memory requirements and massive computations are the main challenges during searches for nearest neighbors.

### Support vector machine (SVM)

The SVM was proposed by Vapnik [38]. The algorithm builds a hyperplane to separate positive and negative samples, and the margin is as large as possible. However, in practice, samples are not linearly separable, and such a hyperplane does not exist. This can lead to poor algorithm performance. Accordingly, the original SVM algorithm is extended for nonlinear classification through the use of kernel functions.

### Random forest (RF)

The RF is established using the numeral of decision trees, and every tree acquires its position arrangement through dissimilar classification [39]. This method permits the evaluation of sampling allocation by using random sampling, which is particularly appropriate for some simple models. The following steps are followed for RF classification.

1. The unique training illustration set is developed, in which the number of cases is *X* and the number of contribution features is *Y*. This illustration is the training set for increasing the tree.
2. A secondary training set is arbitrarily created through sampling with the substitution bootstrap technique for *n* tree times; hence, the subordinate training set for the RF with numeral *n* tree is created.
3. Ahead before the selection of characters (features) for every nonleaf node (internal node), this technique randomly chooses a definite number of characteristics from all distinctiveness, uses them as division characteristics of the existing decision tree, and chooses the optimal one to divide nodes. The number of characters attempted at every division is indicated by mtry, mtry ≤ M.
4. After pruning is considered, the tree expansion is increased.
5. The created trees are joined with an RF. Every tree in the RF transmits an entity choice for the mainly accepted group, and the classifier result is resolute by a mass choice of the trees.

### Gradient boosting decision tree (GBDT)

The boosting method based on gradient descent and its corresponding model are called gradient boosting machines (GBMs) [40]. GBMs construct basic learners through repeated calculations by weighting misclassified observations. The prediction model is an ensemble of weak prediction models. GBMs determine weights by operating the negative partial derivative of the loss function in each training observation. In GBMs, a decision tree is the most common type of weak model used (i.e., gradient boosting decision tree (GBDT)). The GBDT is a model based on a phased manner and can be optimized based on the differentiable loss function. The GBDT uses a fixed-size regression tree as the basic model and uses an iterative calculation method to minimize the loss function. Each regression tree uses the residual of the previous tree to select features and segmentation points, and it sums the outputs of all regression trees as a trained GBDT model.

### eXtreme gradient boosting (XGBoost)

The XGBoost was proposed by Chen and Guestrin in 2016 [41]. XGBoost is an ensemble learning algorithm based on gradient boosting. It provides state-of-the-art results for many bioinformatics problems. XGBoost is essentially an ensemble method based on the gradient boosted tree. The result of the prediction is the sum of scores predicted by trees, as shown in the following equation:

11 $$\hat{y}_{i} = \mathop \sum \limits_{k = 1}^{K} f_{k} \left( {x_{i} } \right), f_{k} \in F$$

where $x_{i}$ is the *i*-th of the training sample, $f_{k} \left( {x_{i} } \right)$ is the score for the *k*-th tree, and *F* is the space of functions containing all gradient boosted trees. The objective function can be optimized using the following equation:

12 $$Obj\left( \theta \right) = \mathop \sum \limits_{i = 1}^{n} l\left( {y_{i} ,\hat{y}_{i} } \right) + \mathop \sum \limits_{k}^{K} \Omega \left( {f_{k} } \right)$$

where $\mathop \sum \limits_{i = 1}^{n} l\left( {y_{i} ,\hat{y}_{i} } \right)$ refers to a differentiable loss function that measures the fitness of model prediction $y_{i}$ and samples of training dataset $\hat{y}_{i}$, and $\mathop \sum \limits_{k}^{K} \Omega \left( {f_{k} } \right)$ is a regularization item that punishes the complexity of the model to avoid overfitting.

### Deep learning (DL)

Deep learning is a branch of ML that uses artificial neural networks to imitate a learning model generated based on the structure of the human brain [42]. The basic unit of an artificial neural network is a neuron. Each neuron is connected to other neurons, can input and output signals, and can transmit information [43]. In the era of big data, DL has been widely used to learn and train models by using large amounts of data to provide future predictions.

The deep neural network (DNN) model is a multilayer perceptron (MLP) neural network that consists of two or more hidden layers and is the basic model of DL [44]. MLP is a feedforward neural network whose architecture consists of an input layer, a hidden layer, and an output layer. Each layer consists of multiple neurons. In the input layer, the neuron takes the input data *X* and transmits this data signal to the next layer of the network. In the next layer, the hidden layer is where each neuron acquires a data signal, which is the weighted sum of the outputs of the neurons in the previous layer. An activation function is applied inside each neuron to control the input. The network applies nonlinear mapping from the input vector to the output, parameterized by weights called the weight vector (*W*). The variables used in DNNs are bias *b*, input *x*, output *y*, weight *w*, calculation function σ, and start function $f\left( \sigma \right)$. Each neuron in a DNN uses the following equation:

17 $$\sigma :Sum = w*x + b$$

18 $$y :f\left( \sigma \right) = f\left( {w*x + b} \right)$$

The input layer is *i* neurons, the hidden layer is *k* layers, the hidden layer is *j* neurons, and the output layer is *x* neurons. The weights between layers are denoted as *W*, and these weights are randomly generated at the beginning of model create. The weights between layers are updated after consideration of the error rate between the model output and actual output. The formula for calculating the number of weights (*W*) between layers is as follows:

19 $$W = \left( {I*H_{1} } \right) + \mathop \sum \limits_{m = 1}^{k - 1} H_{m} *H_{m + 1} + \mathop \sum \limits_{m = 1}^{k} BiasH_{m} + \left( {H_{k} *O} \right) + BiasO$$

**Fig. 3:**
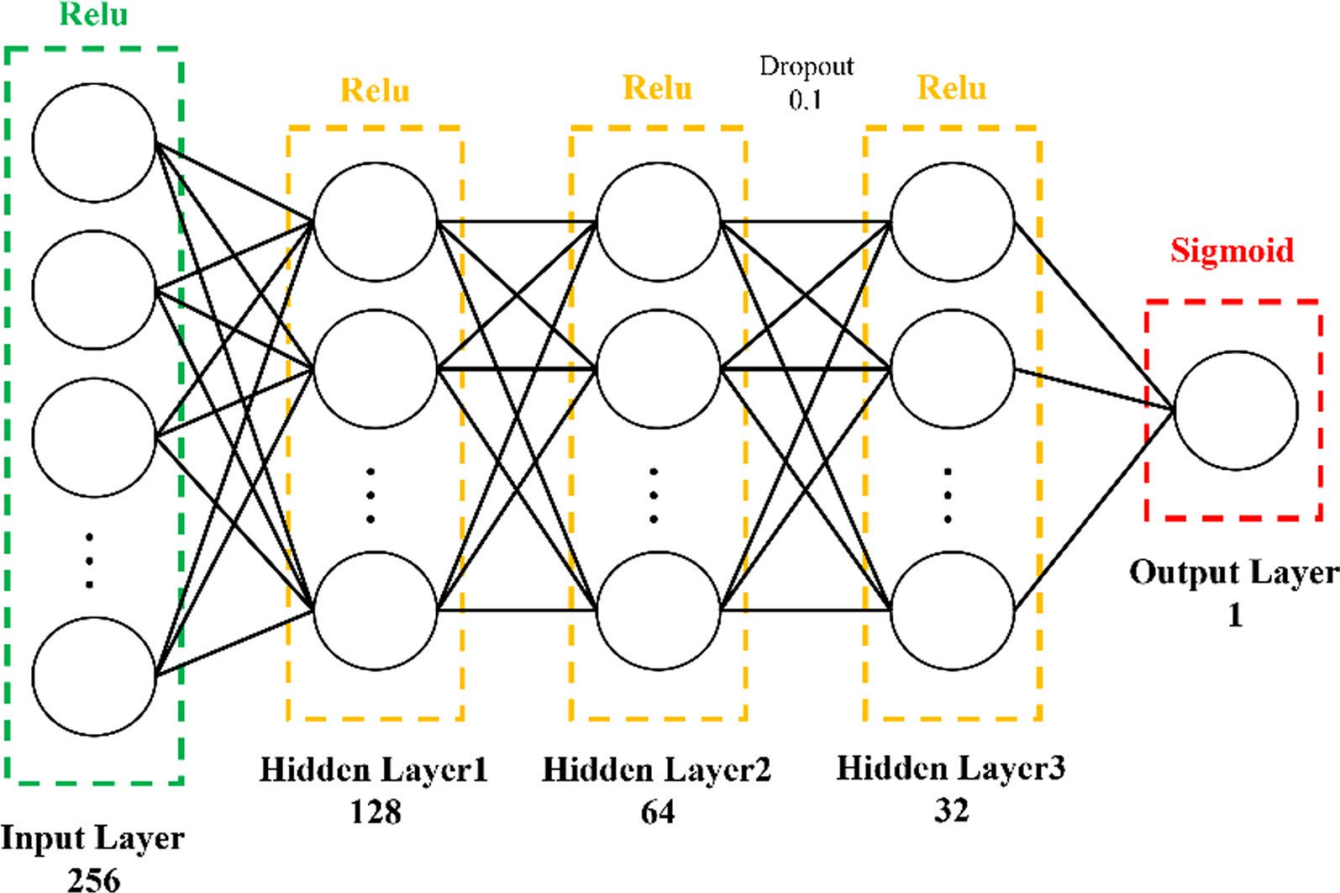
Architecture of the DNN

The MLP algorithm used in this study consisted of one input layer, three hidden layers, and one output layer. Both the input and hidden layers were used a rectified linear unit (ReLu) activation function, and the dropout probability was 0.1 before the last hidden layer. Because a classification problem was examined in this study, the output layer was used as a nonlinear sigmoid activation function. The ReLu and sigmoid activation function formulas are presented as follows, And Fig. 3 presents the DNN architecture.

20 $$\sigma \left( x \right) = \left\{ {\begin{array}{*{20}l} {\max \left( {0,x} \right), } \hfill & {x \ge 0} \hfill \\ {0,} \hfill & {x < 0} \hfill \\ \end{array} } \right.$$

21 $$f\left( z \right) = \frac{1}{{1 + e^{ - z} }}$$

### Bidirectional long short-term memory (Bi-LSTM)

**Fig. 4:**
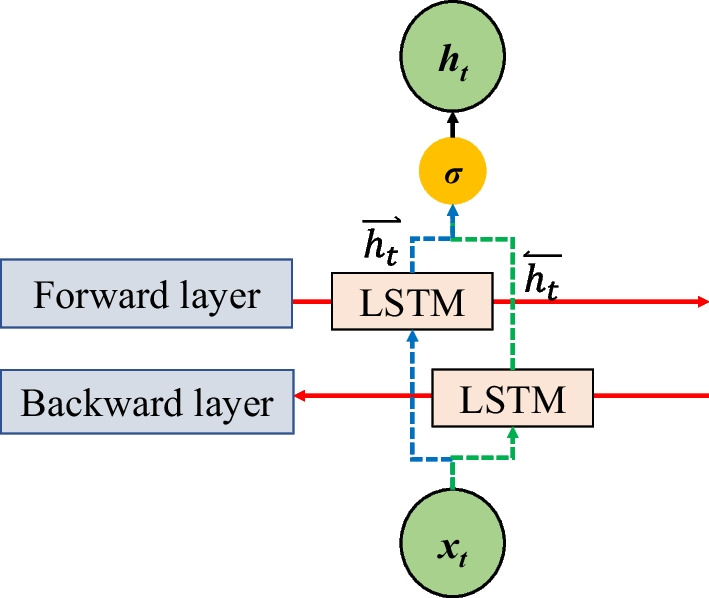
Bi-LSTM architecture

The LSTM employs three custom-built gates to store information [45]. The original architecture is proposed by Hochreiter [46], the update of the cell output state is related to the previous hidden layer output and the current input. Moreover, Hochreiter attached a peephole connection and used the previous cell state as a parameter. For a single LSTM cell, data flow between gates and inputs is depicted in Fig. 4. At each time *t*, $x_{t}$ is the current input, h_t−1_ is the previous hidden state, and $c_{t - 1}$ is the previous cell output state. The outputs of three gates can be calculated using Eqs. (22)–(24). The forget gate $f_{t}$ decides if $c_{t - 1}$ is retained, the input gate decides if the state is updated by the current input $x_{t}$, and the output gate $o_{t}$ decides if $h_{t - 1}$ is passed to the next cell. At each timestamp *t*, $a_{t}$ is the candidate for updating the memory cell. The output of the current LSTM cell $c_{t}$ and the current hidden state $h_{t}$ can be calculated according to Eqs. (25)–(27).

22 $$i_{t} = \sigma \left( {X_{i} x_{t} + H_{i} h_{t - 1} + C_{i} c_{t - 1} + b_{i} } \right)$$

23 $$o_{t} = \sigma \left( {X_{o} x_{t} + H_{o} h_{t - 1} + C_{o} c_{t - 1} + b_{o} } \right)$$

24 $$f_{t} = \sigma \left( {X_{f} x_{t} + H_{f} h_{t - 1} + C_{f} c_{t - 1} + b_{f} } \right)$$

25 $$a_{t} = \sigma \left( {X_{a} x_{t} + H_{a} h_{t - 1} + C_{a} c_{t - 1} + b_{a} } \right)$$

26 $$c_{t} = f_{t} *c_{t - 1} + i_{t} *a_{t}$$

27 $$h_{t} = o_{t} *tanh\left( {c_{t} } \right)$$

In these equations, * represents the element-wise multiplication operator, *H* and *C* are the weights, and *b* are biases.

#### Model evaluation

The data set was randomly divided into two groups: 70% for the training set and 30% for the testing set. We used the training data set to establish a prediction model.

In the binary classification model, the predicted results were combined with actual results to produce four elements, namely true positives, false positives, true negatives, and false negatives, which are represented by TP, FP, TN, and FN respectively (*T* represents a correct prediction and *F* represents an incorrect prediction). This process enables the formation of a confusion matrix using the following formula [47]:

28 $$TPR = \frac{TP}{{TP + FN}}$$

29 $$FPR = \frac{FP}{{FP + TN}}$$

30 $$FNR = \frac{FN}{{TP + FN}}$$

31 $$TNR = \frac{TN}{{FP + TN}}$$

The performance of the models was evaluated using criteria, namely accuracy, prevalence, sensitivity, specificity, the area under the curve (AUC). The area under the ROC curve was used to evaluate the model with the highest accuracy and calculate AUC [48]. The larger the AUC value is, the higher the accuracy is. The relevant equations are as follows:

32 $$Specificity = TNR$$

33 $$Sensitivity = TPR$$

34 $$Prevalence = \frac{TP + FP}{{TP + TN + FP + FN}}$$

35 $$Accuracy = \frac{TP + TN}{{TP + FP + FN + TN}}$$

#### Statistical analysis

Table 1 summarized the demographic characteristics of the patients on HD and the distribution of albumin-related biomarkers, including the mean (standard deviation), frequency (percentage), and median (interquartile range). Differences between the patients with a 3-month mean albumin level of ≥ 3.5 g/dL and those with a 3-month mean albumin level of < 3.5 g/dL were determined using independent two-sample t-tests or chi-squared tests, as appropriate. Pearson’s correlation analysis was performed, and correlation plots and correlation heatmaps were drawn to assess collinearity between mean albumin levels and biomarkers.

**Table 1:** Baseline characteristics of 3-months mean albumin in new HD patients were divided into 2 categories (n = 1567)

Variables	Total	Mean albumin ≥ 3.5 g/dL	Mean albumin < 3.5 g/dL	*P*
Case no. (row%)	1567 (100.0%)	1283 (81.9%)	284 (18.1%)	
Age (years)	67.0 ± 13.9	65.4 ± 13.5	74.2 ± 13.2	**< 0.001**
Gender				**0.002**
Female	718 (45.8%)	564 (44%)	154 (54.2%)	
Male	849 (54.2%)	719 (56%)	130 (45.8%)	
Comorbidity				
Diabetes mellitus				**0.015**
	822 (52.5%)	654 (51%)	168 (59.2%)	
Hypertension				0.198
	1262 (80.5%)	1025 (79.9%)	237 (83.5%)	
Heart Failure				**0.030**
	326 (20.8%)	253 (19.7%)	73 (25.7%)	
Caner				**0.003**
	545 (34.8%)	424 (33%)	121 (42.6%)	
Mortality				**< 0.001**
Alive	1497 (95.5%)	1254 (97.7%)	243 (85.6%)	
Dead	70 (4.5%)	29 (2.3%)	41 (14.4%)	
Laboratory measurements				
Hb, g/dL	10.5 ± 1.3	10.6 ± 1.2	9.9 ± 1.5	**< 0.001**
Albumin, g/dL	3.8 ± 0.4	3.9 ± 0.3	3.2 ± 0.4	**< 0.001**
Fe, ug/dL	68.8 ± 29.5	70.1 ± 27	63.1 ± 38.5	**0.004**
Ferritin, ug/dL	479.7 ± 523.3	426.9 ± 395.3	718.3 ± 858.9	**< 0.001**
Na, mEq/L	136.3 ± 3.3	136.5 ± 3.1	135.4 ± 3.8	**< 0.001**
K, mEq/L	4.5 ± 0.7	4.6 ± 0.7	4.3 ± 0.7	**< 0.001**
Ca, mg/dL	9.2 ± 0.8	9.3 ± 0.8	9.0 ± 0.9	**< 0.001**
Phosphate, mg/dL	5.0 ± 1.4	5.1 ± 1.4	4.5 ± 1.5	**< 0.001**
BUN, mg/dL	66.7 ± 19.9	67.5 ± 18.7	62.7 ± 24.5	**0.002**
Cr, mg/dL	10.1 ± 2.7	10.5 ± 2.6	8.0 ± 2.6	**< 0.001**
Alkaline phosphatase, U/L	83.4 ± 52.4	77.6 ± 42.1	109.3 ± 79.6	**< 0.001**
iPTH, pg/dL	207.6 (97.7–408.3)	219.8 (105.9–423.6)	163.7 (67.7–323.5)	**0.004**
Cholesterol, mg/dL	163.6 ± 38.8	165.7 ± 38.0	153.8 ± 40.9	**< 0.001**
Triglyceride, mg/dL	156.5 ± 121.2	160.3 ± 124.6	139.5 ± 103.1	**0.003**
Fasting glucose (AC), mg/dL	142.5 ± 77.3	139.4 ± 74.6	156.2 ± 87.3	**0.003**

Boldface was considered statistically significant (*P* value < 0.05)

*Hb* hemoglobin, *Na* sodium, *K* potassium, *Ca* calcium, *BUN* blood urea nitrogen, *Cr* creatinine, *iPTH* intact parathyroid hormone

Associations between mean albumin levels and individual factors were analyzed using univariate logistic regression analysis. Multivariate logistic regression was used to analyze associations between mean albumin categories and multiple factors. The full adjusted model included all factors, whereas the GOA model selected factors by using the GOA. Odds ratios (ORs) and 95% confidence intervals (CIs) were calculated. The performance of multiple logistic regression models was compared based on the Akaike information criterion (AIC). A low AIC value indicated a low prediction error for the corresponding model. The g-computation method was used to calculate the factor weights. These weights were used to adjust the original blood value and highlight the importance of factors. The SMOTE method was used to solve the data imbalance problem. All P values were two-tailed, and a *P* value of < 0.05 was considered statistically significant. All statistical analyses were performed using R version 4.0.5 (R Development Core Team 2022). The relevant packages used are as follows: stats, My.stepwise, metaheuristicOpt, e1071, keras, tensorflow, etc.

## Results

### Baseline characteristics and laboratory measurement distributions of patients on HD

Table 1 presented the distribution of clinicopathological characteristics between the patients with a 3-month mean albumin level of ≥ 3.5 g/dL and those with a 3-month mean albumin level of < 3.5 g/dL. Among the 1567 patients on HD included in this study, 1283 and 284 had 3-month mean albumin levels of ≥ 3.5 and < 3.5 g/dL, respectively. The patients on HD with a 3-month mean albumin level of < 3.5 g/dL were older, had a higher prevalence of diabetes mellitus and heart failure, and a higher risk of mortality. Moreover, the laboratory measurements significantly different between the groups.

### Individual factors affecting mean 3-month albumin levels

Table 2 presented the results of the univariate logistic regression analysis of mean 3-month albumin levels before death in patients on HD. The results revealed that older age (OR = 1.05, 95% CI = 1.04–1.06, *P* < 0.001), diabetes mellitus (OR = 1.39, 95% CI = 1.07–1.81, *P* = 0.013), heart failure (OR = 1.41, 95% CI = 1.04–1.89, *P* = 0.025), and cancer (OR = 1.50, 95% CI = 1.16–1.95, *P* = 0.002) were associated with a mean 3-month albumin level of < 3.5 g/dL. In terms of laboratory measurements, low hemoglobin levels (OR = 0.63, 95% CI = 0.57–0.70, *P* < 0.001), low Fe levels (OR = 0.99, 95% CI = 0.99–1.00, *P* < 0.001), high ferritin levels (OR = 1.001, 95% CI = 1.0007–1.0012, *P* < 0.001), low sodium levels (OR = 0.90, 95% CI = 0.87–0.94, *P* < 0.001), low potassium levels (OR = 0.54, 95% CI = 0.44–0.66, *P* < 0.001), low calcium levels (OR = 0.61, 95% CI = 0.51–0.72, *P* < 0.001), low phosphate levels (OR = 0.70, 95% CI = 0.63–0.77, *P* < 0.001), low blood urea nitrogen levels (OR = 0.99, 95% CI = 0.89–0.99, *P* < 0.001), low creatinine levels (OR = 0.67, 95% CI = 0.63–0.71, *P* < 0.001), high alkaline phosphatase levels (OR = 1.01, 95% CI = 1.0073–1.0124, *P* < 0.001), and low cholesterol levels (OR = 0.99, 95% CI = 0.99–1.00, *P* < 0.001) were associated with a 3-month mean albumin level of < 3.5 g/dL. The first blood values 3 months prior to death of patients had significant associations with the mean albumin levels in the 3 months prior to death according to a univariate analysis.

**Table 2:** Regression analysis for 3-months albumin mean univariate logistic (n = 1567)

Characteristics	Comparison	Unadjusted		
		OR	95% CI	*P*
Age	Years	1.05	1.04–1.06	**< 0.001**
Gender	Male v.s Female	0.66	0.51–0.86	**0.002**
Comorbidity				
Diabetes mellitus	Yes v.s No	1.39	1.07–1.81	**0.013**
Hypertension	Yes v.s No	1.27	0.91–1.8	0.171
Heart Failure	Yes v.s No	1.41	1.04–1.89	**0.025**
Cancer	Yes v.s No	1.50	1.16–1.95	**0.002**
Laboratory measurements				
Hb	g/dL	0.63	0.57–0.70	**< 0.001**
Fe	ug/dL	0.99	0.99–1.00	**< 0.001**
Ferritin	ug/dL	1.001	1.0007–1.0012	**< 0.001**
Na	mEq/L	0.90	0.87–0.94	**< 0.001**
K	mEq/L	0.54	0.44–0.66	**< 0.001**
Ca	mg/dL	0.61	0.51–0.72	**< 0.001**
Phosphate	mg/dL	0.70	0.63–0.77	**< 0.001**
BUN	mg/dL	0.99	0.98–0.99	**< 0.001**
Cr	mg/dL	0.67	0.63–0.71	**< 0.001**
Alkaline phosphatase	U/L	1.01	1.0073–1.0124	**< 0.001**
iPTH	pg/dL	0.999	0.9987–0.9997	**0.004**
Cholesterol	mg/dL	0.99	0.99–1.00	**< 0.001**
Triglyceride	mg/dL	0.998	0.9969–0.9995	**0.009**
Fasting glucose (AC)	mg/dL	1.002	1.001–1.004	**0.001**
Optimal AIC		**1272.75**		

Boldface was considered statistically significant (*P* value < 0.05)

### Multifactorial influencing factors of mean 3-months albumin levels determined using GOA

Table 3 summarized the results of the multivariate logistic regression analysis on mean albumin levels in new patients on HD 3 months prior to death, obtained using the fully adjusted model and the GOA feature selection model. Older age (OR = 1.01, 95% CI = 1.01–1.04, *P* < 0.001), low iron levels (OR = 0.99, 95% CI = 0.98–0.99, *P* < 0.001), low creatinine levels (OR = 0.77, 95% CI = 0.71–0.84, *P* < 0.001), and high alkaline phosphatase levels (OR = 1.01, 95% CI = 1.00–1.01, *P* < 0.001) were determined to be significant in the fully adjusted logistic regression model. Feature selection was performed using the GOA to select 12 out of 20 clinical factors, namely age; gender; hypertension; and hemoglobin, iron, ferritin, sodium, potassium, calcium, creatinine, alkaline phosphatase, and triglyceride levels. Older age (OR = 1.03, 95% CI = 1.02–1.04,* P* < 0.001), male (OR = 1.48, 95% CI = 1.07–2.06, *P* = 0.018), low hemoglobin levels (OR = 0.83, 95% CI = 0.73–0.95, *P* = 0.006), low iron levels (OR = 0.99, 95% CI = 0.99–1.00, *P* < 0.001), high ferritin levels (OR = 1.001, 95% CI = 1.0004–1.0011, *P* < 0.001), low Na levels (OR = 0.94, 95% CI = 0.90–0.98, *P* = 0.005), low K levels (OR = 0.79, 95% CI = 0.64–0.98, *P* = 0.037), low Ca levels (OR = 0.72, 95% CI = 0.59–0.86, *P* = 0.001), low creatinine levels (OR = 0.77, 95% CI = 0.71–0.83, *P* < 0.001), high alkaline phosphatase levels (OR = 1.01, 95% CI = 1.00–1.01, *P* < 0.001), and low triglyceride levels (OR = 0.998, 95% CI = 0.9968–0.9998, *P* = 0.030) were all significant in the GOA feature selection model. The best AIC of the fully adjusted logistic regression model and the GOA feature selection model were 1173.52 and 1160.71, respectively. The results indicated that the GOA feature selection model had a lower AIC and a higher accuracy in selecting risk factors for the low serum albumin.

**Table 3:** Regression analysis for 3-months albumin mean multivariate logistic (n = 1567)

Characteristics	Comparison	Fully adjusted			GOA feature selection		
		OR	95% CI	*P*	OR	95% CI	*P*
Age	Years	1.03	1.01–1.04	** < 0.001**	1.03	1.02–1.04	**< 0.001**
Gender	Male v.s Female	1.41	1.00–1.99	0.051	1.48	1.07–2.06	**0.018**
Comorbidity							
Diabetes mellitus	Yes v.s No	0.85	0.59–1.2	0.356			
Hypertension	Yes v.s No	1.05	0.70–1.60	0.805	1.01	0.68–1.52	0.965
Heart Failure	Yes v.s No	1.05	0.74–1.49	0.778			
Cancer	Yes v.s No	1.14	0.84–1.56	0.389			
Laboratory measurements							
Hb	g/dL	0.85	0.74–0.96	**0.012**	0.83	0.73–0.95	**0.006**
Fe	ug/dL	0.99	0.98–0.99	** < 0.001**	0.99	0.98–0.99	**< 0.001**
Ferritin	ug/dL	1.001	1.0003–1.0010	** < 0.001**	1.001	1.0004–1.0011	**< 0.001**
Na	mEq/L	0.94	0.90–0.98	**0.005**	0.94	0.90–0.98	**0.005**
K	mEq/L	0.79	0.62–0.99	**0.045**	0.79	0.64–0.98	**0.037**
Ca	mg/dL	0.71	0.59–0.86	**0.001**	0.72	0.59–0.86	**0.001**
Phosphate	mg/dL	0.99	0.87–1.13	0.873			
BUN	mg/dL	1.00	0.99–1.01	0.951			
Cr	mg/dL	0.77	0.71–0.84	** < 0.001**	0.77	0.71–0.83	**< 0.001**
Alkaline phosphatase	U/L	1.01	1.00–1.01	** < 0.001**	1.01	1.00–1.01	**< 0.001**
iPTH	pg/dL	1.00	0.9994–1.0005	0.951			
Cholesterol	mg/dL	0.997	0.99–1.00	0.259			
Triglyceride	mg/dL	0.999	0.9971–1.0003	0.133	0.998	0.9968–0.9998	**0.030**
Fasting glucose (AC)	mg/dL	1.00	0.9981–1.0022	0.885			
Optimal AIC		**1173.52**			**1160.71**		

Boldface was considered statistically significant (*P* value < 0.05)

*GOA* Grasshopper Optimization Algorithm, *AIC* Akaike information criterion

### Quantile g-computation adjustment of factor weights

**Fig. 5:**
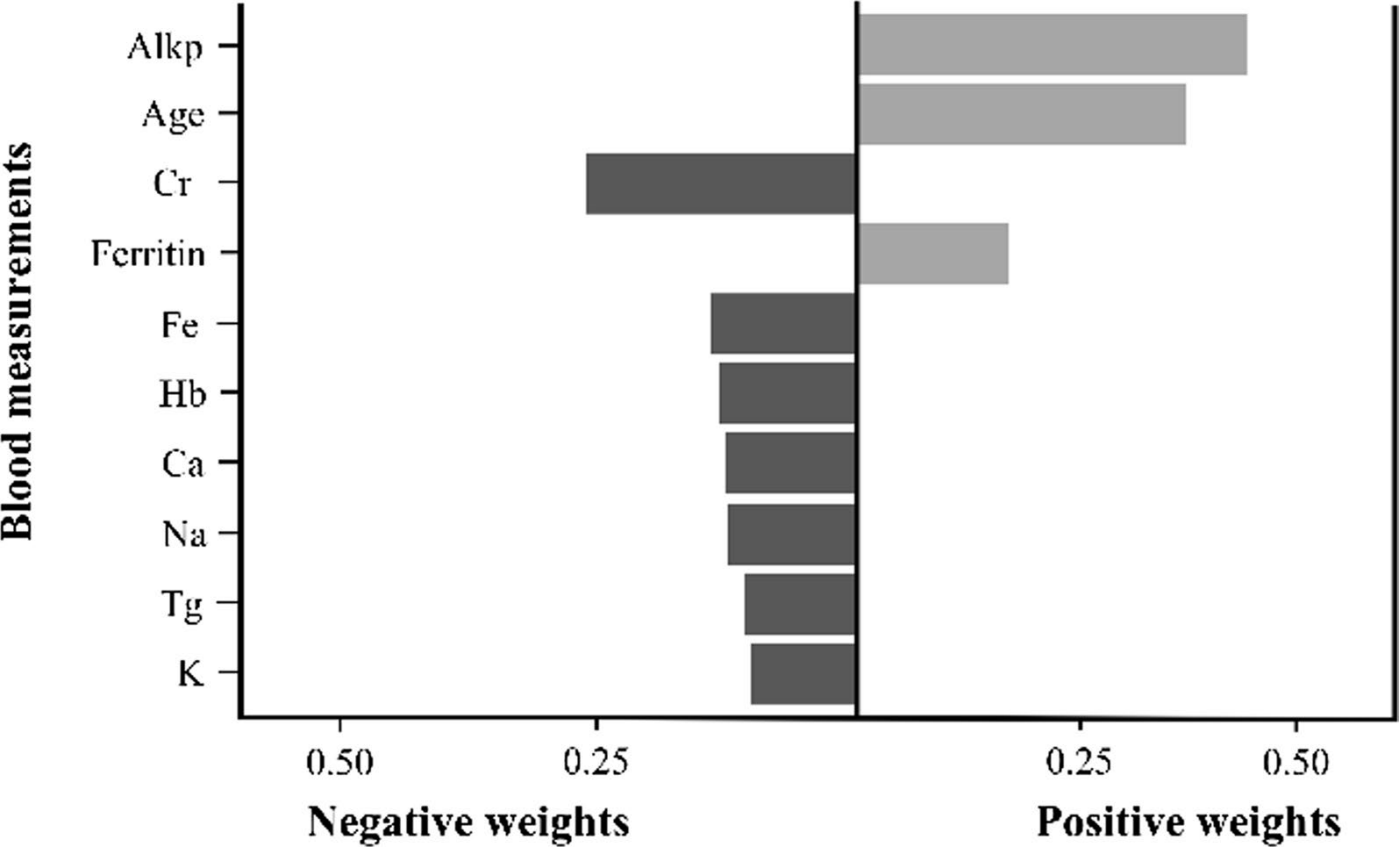
Weights representing the proportion of the positive or negative partial effect of biomarkers selected using the GOA in the quantile g-computation method

Figure 5 presented the risk factors for the low serum albumin selected using the GOA, and the weight ratio of each factor was calculated using the quantile g-computation method. Alkaline phosphatase was assigned the highest positive weight, followed by age and ferritin levels. Creatinine was assigned the largest negative weight, followed by blood measurements such as iron and hemoglobin levels. In addition, age and creatinine levels were identified as more crucial risk factors for low serum albumin levels than other clinical factors.

### Prediction of the low serum albumin

**Fig. 6:**
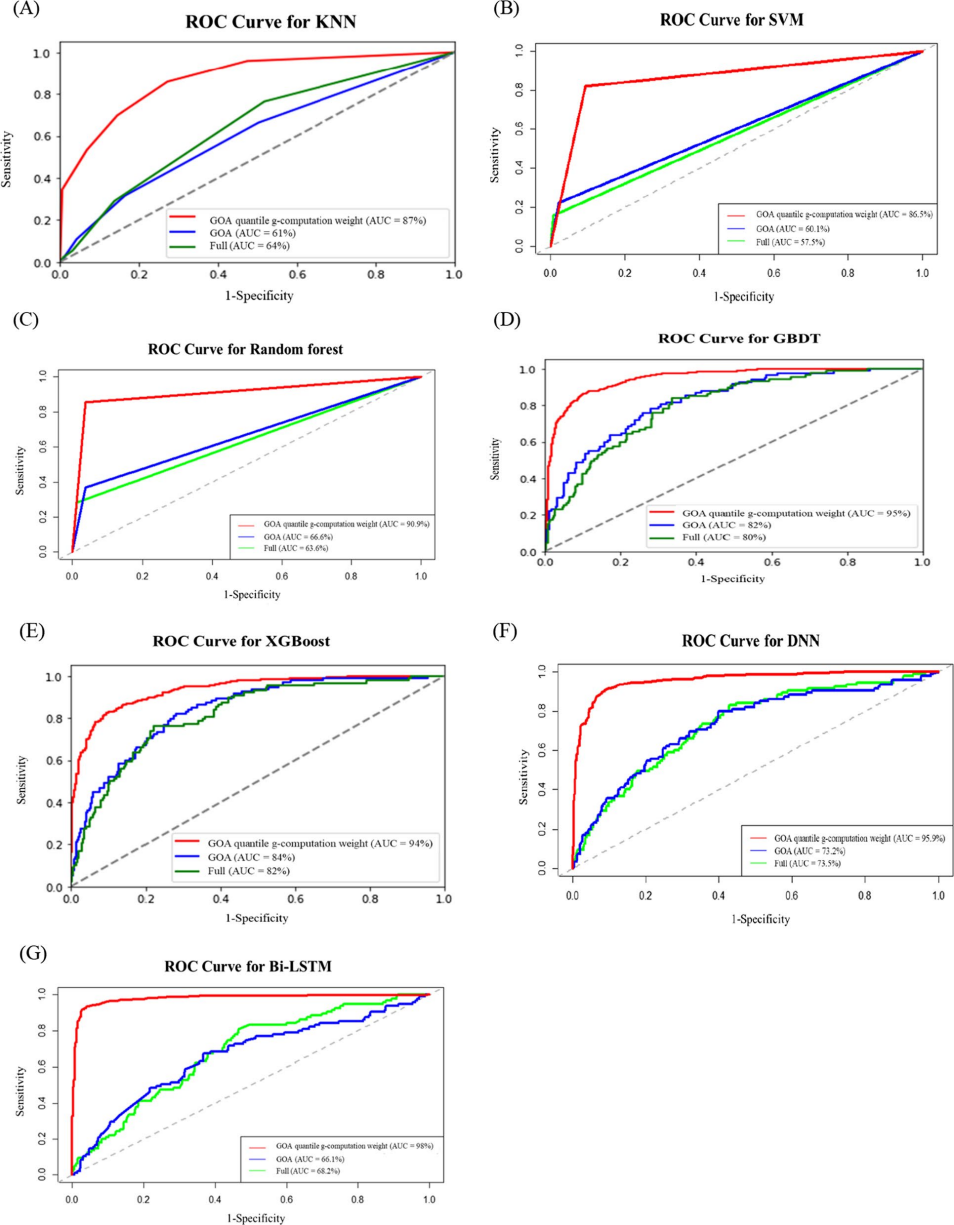
ROC curves for the **A** KNN, **B** SVM, **C** RF, **D** GBDT, **E** XGBoost, **F** DNN, and **G** Bi-LSTM methods

In this study, we used three models and seven methods to predict the low serum albumin. The three models were the fully adjusted, GOA, and GOA quantile g-computation weight models. Subsequently, we compared the prediction performance of the three models by using seven methods, namely KNN, SVM, RF, GBDT, XGBoost, DNN, and Bi-LSTM. We examined the predictive performance of the three models by using the seven methods based on their accuracy, prevalence, sensitivity, specificity, and AUC (Table 4 and Fig. 6). Table 4 presented the prediction results for the three models. The results revealed that the seven methods predicted the performance of the three models, respectively, and the accuracy and AUC of the GOA quantile g-computation weight model were higher than those of the other two models. Compared with the GOA model, the accuracy of the GOA quantile g-computation weight model improved by 0.1, 0.3, 0.6, 0.3, 0.5, and 0.12 when the KNN, SVM, RF, GBDT, XGBoost, and DNN methods were used, respectively. However, compared with the fully adjusted model and GOA model, the accuracy of the Bi-LSTM combined with the GOA quantile g-computation weight model improved by at least 0.16 and at the most by 0.21. The Bi-LSTM method combined with the GOA quantile g-computation weight model yielded the most favorable results for predicting the low serum albumin. In order to prove the performance of the proposed model objective, the data set was cut five times using cross-validation, and the average results are shown in Table 5.

**Table 4:** Comparison of the prediction performance of 3-months albumin average with 2 categories

Method	Model	Accuracy	Prevalence	Sensitivity	Specificity	AUC
KNN	Full	0.79	0.20	0.05	0.97	0.64
	GOA	0.79	0.20	0.11	0.96	0.61
	GOA quantile g-computation weight	0.80	0.36	0.70	0.85	0.87
SVM	Full	0.83	0.19	0.16	0.99	0.58
	GOA	0.85	0.16	0.22	0.98	0.60
	GOA quantile g-computation weight	0.88	0.37	0.82	0.91	0.86
RF	Full	0.85	0.19	0.23	0.99	0.64
	GOA	0.86	0.17	0.37	0.96	0.67
	GOA quantile g-computation weight	0.92	0.36	0.87	0.96	0.91
GBDT	Full	0.82	0.19	0.24	0.95	0.80
	GOA	0.85	0.17	0.28	0.97	0.82
	GOA quantile g-computation weight	0.88	0.36	0.78	0.94	0.95
XGBoost	Full	0.83	0.19	0.24	0.96	0.82
	GOA	0.83	0.20	0.30	0.96	0.84
	GOA quantile g-computation weight	0.88	0.35	0.79	0.93	0.94
DNN	Full	0.78	0.20	0.29	0.91	0.74
	GOA	0.79	0.20	0.24	0.93	0.73
	GOA quantile g-computation weight	0.91	0.36	0.87	0.94	0.96
Bi-LSTM	Full	0.74	0.20	0.24	0.86	0.68
	GOA	0.76	0.20	0.15	0.95	0.66
	GOA quantile g-computation weight	0.95	0.36	0.92	0.97	0.98

**Table 5:** Comparison of the prediction performance of GOA quantile g-computation weight with 5 cross-validation

Times	Accuracy	Prevalence	Sensitivity	Specificity	AUC
1	0.93	0.37	0.91	0.95	0.98
2	0.94	0.37	0.89	0.96	0.98
3	0.96	0.38	0.95	0.96	0.99
4	0.95	0.37	0.95	0.95	0.98
5	0.94	0.38	0.92	0.95	0.98

Figure 6 presented a comparison of the ROC curves of the seven methods for the three models. The results revealed that the AUC of the GOA quantile g-computation weight model was higher than that of the other two models. The seven methods with the GOA quantile g-computation weight model were used to obtain AUC values. The AUC values obtained using the KNN, SVM, RF, GBDT, XGBoost, DNN, and Bi-LSTM methods were 0.87, 0.86, 0.91, 0.95, 0.94, 0.96, and 0.98, respectively. Moreover, the results revealed that the prediction performance of the Bi-LSTM method combined with the GOA quantile g-computation weight model was significantly higher than that of the other methods.

### Correlations between biomarkers and serum albumin

**Fig. 7:**
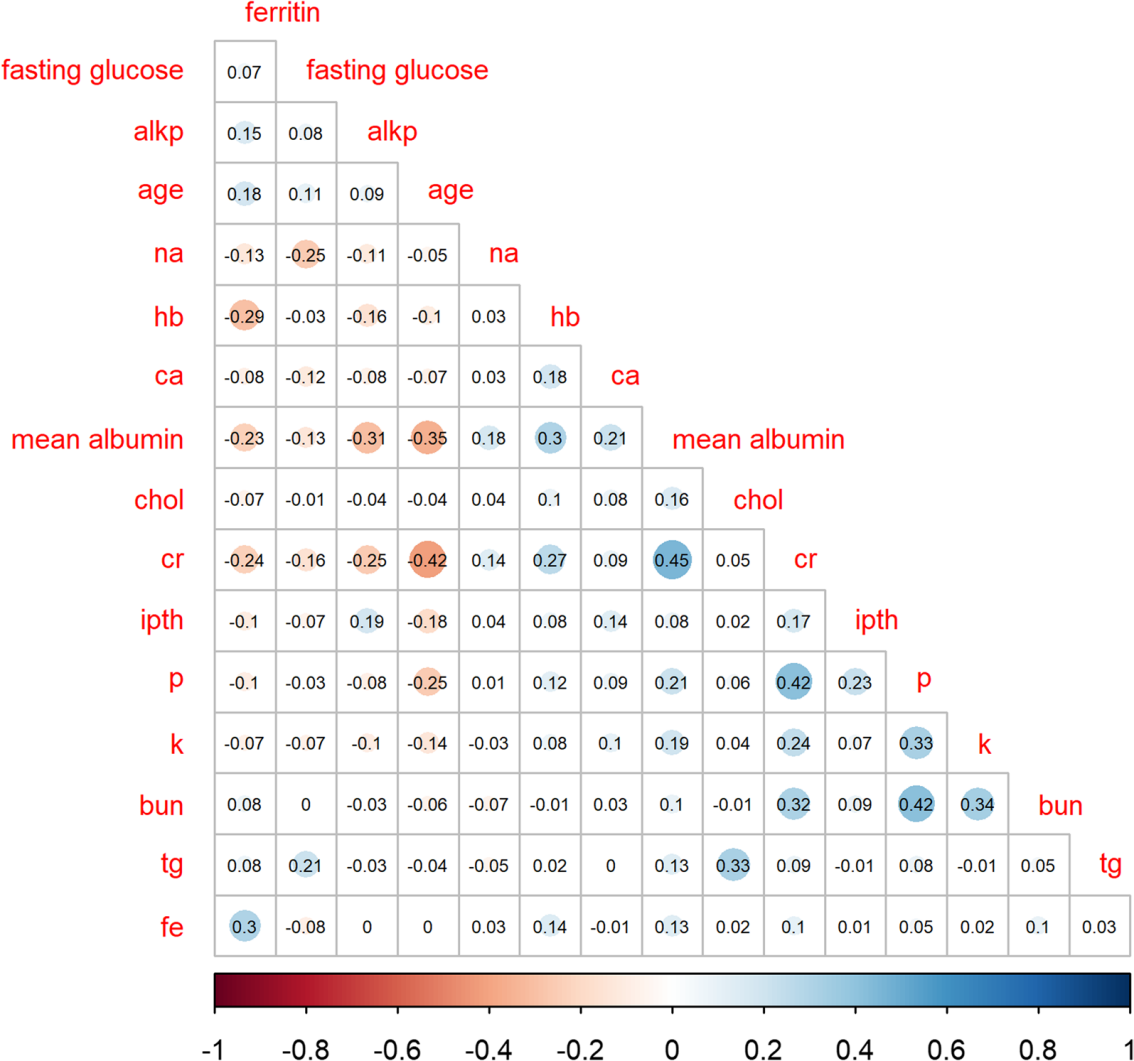
Pearson correlations between studied biomarkers and serum albumin levels

Figure 7 presented a heatmap depicting the correlation between serum albumin levels and 15 biomarkers. The saturation and size of the circle indicate the magnitude of correlations. Blue indicates a positive correlation, and red indicates a negative correlation.

Strong positive correlations were observed between mean albumin and creatinine levels [49], between creatinine and phosphate levels [50], and between phosphate and blood urea nitrogen levels.

Strong negative correlations were observed between age and creatinine levels, between age and mean albumin levels, and between alkaline phosphatase and mean albumin levels.

In summary, positive and negative correlations were noted between the biomarkers. The factors with strong correlations were related to nutritional status and clinical significance [51]. For example, advanced age may affect basal metabolism and nutrient absorption, and creatinine is mainly related to metabolites released due to muscle activity. For patients on HD, dietary control is crucial to health. Phosphate is obtained from the human diet, and its intake should be balanced.

## Discussion

This study used data from the longitudinal electronic health records of the largest HD center in Taiwan. Many studies have reported that serum albumin level is a nutritional indicator for HD, and previous studies using long-term clinical data have demonstrated a relationship between hypoalbuminemia and mortality in patients on HD [52, 53]. In this study, we observed that the albumin levels of Taiwanese patients receiving maintenance HD was unstable 3 months before death, and their albumin value was mostly less than the normal value of 3.5 g/dL. Therefore, in this study, we used the DL method to predict whether the mean albumin level of patients on HD was low 3 months before their death, and the first measurement obtained 3 months before death was used to predict the low serum albumin. The results of this study indicated that the use of the GOA quantile g-computation weight model combined with the DL method can improve the efficiency of clinical factor screening and the accuracy of the low serum albumin prediction.

### Principal results

A complex interaction exists between clinical biomarkers. The findings of preliminary analysis in this study revealed that the 3-month mean albumin level in patients on HD was 3.8 ± 0.4 g/dL. Furthermore, the 3-month mean albumin level before the end of the study follow-up and before death were 3.8 ± 0.4 and 3.4 ± 0.5, respectively, and the levels did not significantly differ between the patients who survived and those who died (*P* < 0.001). The 3-month mean albumin level before death was correlated with mortality. This study identified risk factors associated with the low serum albumin. The results of univariate logistic regression analysis revealed that the first three laboratory values of the patients on HD before death were significantly correlated with their albumin level in the 3 months before death. Furthermore, the findings of multivariate logistic regression analysis indicated that the factors determined to be significantly correlated with albumin level in the univariate model exhibited nonsignificant correlations in the fully adjusted multivariate model; this finding might be due to interactions among factors. Therefore, we used the GOA feature selection method to identify crucial risk factors for the low serum albumin. The advantage of the GOA feature selection method is its high compatibility and its ability to accelerate convergence to provide a global optimal solution. Using the GOA for feature selection, we selected 12 out of 20 clinical factors, namely age; gender; hypertension; and hemoglobin, iron, ferritin, sodium, potassium, calcium, creatinine, alkaline phosphatase, and triglyceride levels; all these factors were significant.

We determined that the women (OR = 0.66, 95% CI = 0.51–0.86, *P* = 0.002) had a significantly higher risk of the low serum albumin in the univariate model, whereas the men had a nonsignificantly higher risk of the low serum albumin in the multivariate fully adjusted model (OR = 1.41, 95% CI = 1.00–1.99, *P* = 0.051). Among the factors selected by the GOA, male (OR = 1.48, 95% CI = 1.07–2.06, *P* = 0.018) was associated with a higher risk of the low serum albumin. Moreover, we observed that a low triglyceride level (OR = 0.999, 95% CI = 0.9971–1.0003, *P* = 0.133) was associated with a higher risk of the low serum albumin in the multivariate fully adjusted model; however, this association was not significant. Similarly, among the factors selected by the GOA model, a low triglyceride level (OR = 0.998, 95% CI = 0.9968–0.9998, *P* = 0.030) was significantly associated with a higher risk of the low serum albumin. The findings indicate that these factors can be used in combination to predict the low serum albumin, and they possibly reflect interactions between biomarkers.

For prediction, this study used three models, namely the fully adjusted, GOA, and GOA g-computation weight models, and seven methods, namely the KNN, SVM, RF, GBDT, XGBoost, DNN and Bi-LSTM. The GOA quantile g-computation weight model used the GOA to select the most favorable combination factors associated with the low serum albumin. Subsequently, the g-computation method was used to calculate the weight of each factor. This weight was used to adjust the original blood value such that the important blood factors have a greater impact on the fitness through the weight adjustment, thus improving the predictive ability of the model. In addition, the problem of data imbalance often occurs when medical data are used. Thus, we used the SMOTE method to solve this problem and subsequently used each of the seven methods to compare the performance of the models. The results revealed no significant differences between the accuracy and AUC of the fully adjusted model and those of the GOA model determined using all the aforementioned seven methods. However, the accuracy and AUC of the GOA quantile g-computation weight model determined using the seven methods in combination were significantly higher than those of the other two models. Moreover, the accuracy and AUC of the GOA quantile g-computation weight model determined using the DL method were higher than those of the other two models. This finding may have arisen because DL involves the simulation of the basic operating principles of the nervous system in the human brain. Thus, with the adjusted value of the weight, coupled with the powerful self-learning ability of the DL method, model constantly recalculates weights and training. The DL method exerted the multiplier effect and improved the prediction ability of model.

### Comparison with prior studies

**Fig. 8:**
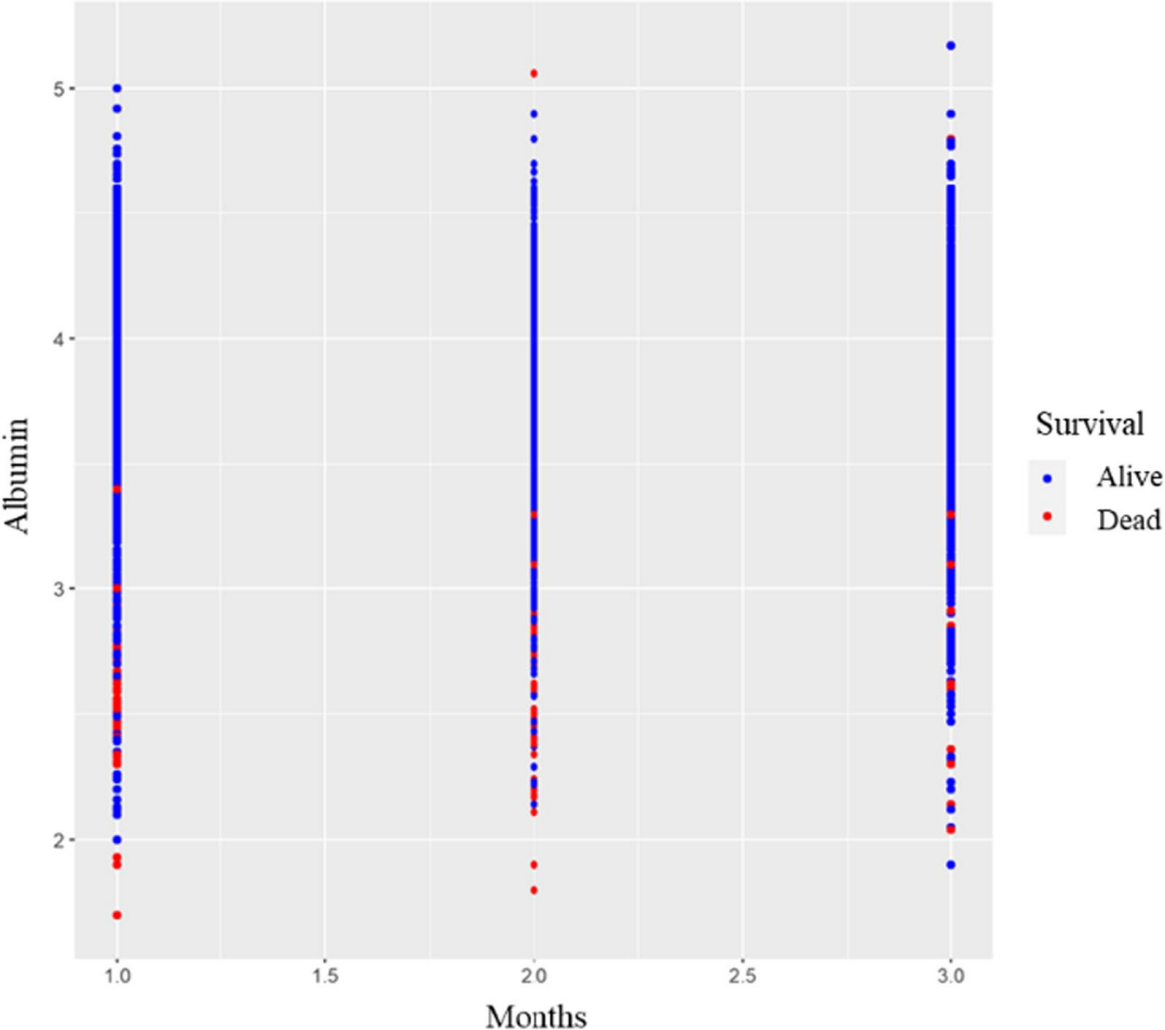
Scatter diagram of the distribution of albumin levels in the 3 months before the death of patients on HD

Hypoalbuminemia in patients on HD is associated with malnutrition, inflammation, and increased mortality [54, 55]. Figure 8 presents the distribution of albumin levels in the 3 months before the death of patients on HD. The dots on the left side represent the distribution of albumin levels 1 month before death, and those on the right side represent the distribution of albumin levels 3 months before death. The blue dots represent the albumin levels of the patients who survived, and the red dots represent the albumin levels of the patients who died. When the distribution line segment in Fig. 8 is viewed from right to left, we can observe that patients who died had lower levels of albumin 3 months before death compared to patients who survived. The middle dots present the distribution of albumin levels 2 months before death. The albumin levels of the patients 2 months before death exhibited a downward trend, and most of these patients eventually had an albumin level of 3 ≤ g/dL. Finally, the distribution of the albumin levels for the month before death of the deceased patient is shown on the far left. The albumin levels of these patients were between 2 and 3 g/dL, and a few extreme values were noted below 2 g/dL. This finding indicated that the low serum albumin is associated with mortality; this result is consistent with those of previous studies.

This study identified and predicted factors associated with the low serum albumin. These factors can be used to predict the mortality risk of patients on HD. We used the GOA quantile g-computation weight model combined with the DL method to determine the optimal combination of factors associated with low serum albumin levels in patients on HD. The related factors included age; gender; hypertension; and hemoglobin, iron, ferritin, sodium, potassium, calcium, creatinine, alkaline phosphatase, and triglyceride levels. According to previous studies and clinical viewpoints, organ failure eventually occurs in older patients, resulting in the impairment of some repair and absorption mechanisms, which may easily lead to malnutrition and indirectly increase the risk of mortality [56, 57]. Patients with chronic kidney disease often experience loss of appetite. Inflammation is highly correlated with appetite, and men have a higher risk of anorexia than have women [55]. Because of differences in body composition between men and women, such as in hormones, muscle mass, and body water content, the severity of related symptoms may be different [56, 57]. Female patients on HD appear to have a survival advantage over male patients on HD because of the presence of sex hormones, which reduce the likelihood of women developing anorexia and malnutrition [58]. In addition, appetite may affect biomarkers and physical indicators, and decreased appetite may lead to decreased concentrations of nutrition-related biomarkers, such as serum albumin and creatinine [55]. Moreover, dialysis concentration may affect dialysis efficacy [59]. A study reported that the dialysis efficacy of patients who died was lower than that of those who survived; lower dialysis efficacy results in lower levels of calcium, creatinine and a lower ultrafiltration volume [60]. Impaired nutritional status results in lower levels of triglycerides, lower levels of density lipoprotein cholesterol, and a lower body mass index [61, 62]. In summary, the optimal factors associated with low serum albumin levels in patients on HD determined using the GOA appeared to be strongly correlated with nutritional status.

### Limitations

This study has some limitations due to its retrospective nature. First, previous studies have reported that albumin indicators are related to nutritional status. This study did not consider patients’ body composition and the discomfort caused by inappropriate dialysis doses. Second, our results may be limited by potential residual confounding effects, such as daily physical activity, dietary intake, and quality of life. Finally, factors associated with the low serum albumin might differ between gender, and this study did not consider gender differences in individual analysis. Previous studies have reported that gender differences affect biomarkers. In this study, we observed that gender affected albumin levels. Therefore, a separate analysis based on gender should be conducted in future studies and can improve the improve clinical care. Furthermore, studies should examine the effects of additional clinical factors on patients on HD, including comorbidities, medication, and dietary intake.

## Conclusions

Malnutrition is often observed in patients receiving long-term HD treatment. Previous studies have reported that the all-cause mortality of patients on HD is related to nutritional status. In this study, the GOA was used to select the factors most associated with the low serum albumin. Because data may be affected by interference factors, we used the quantile g-computation method to calculate the weights for adjustment. Finally, we used the DL method to determine the most effective prediction model. The GOA selected 12 parameters, namely age; gender; hypertension; and hemoglobin, iron, ferritin, sodium, potassium, calcium, creatinine, alkaline phosphatase, and triglyceride levels, which were significantly associated with the low serum albumin. By selecting factors through the GOA and using the quantile g-computation method for weight adjustment in combination with the DL method, we determined the most effective prediction model. The GOA quantile g-computation weight model combined with the DL method can help in accurately predicting the low serum albumin in new patients on HD. The selected factors should be considered for further nutritional management of patients on HD. Appropriate prognostic care and treatment are essential for improving the quality of life of patients on HD.

## Data Availability

Not applicable.
